# Random Telegraph Noises from the Source Follower, the Photodiode Dark Current, and the Gate-Induced Sense Node Leakage in CMOS Image Sensors †

**DOI:** 10.3390/s19245447

**Published:** 2019-12-10

**Authors:** Calvin Yi-Ping Chao, Shang-Fu Yeh, Meng-Hsu Wu, Kuo-Yu Chou, Honyih Tu, Chih-Lin Lee, Chin Yin, Philippe Paillet, Vincent Goiffon

**Affiliations:** 1 Taiwan Semiconductor Manufacturing Company (TSMC), Hsinchu 30077, Taiwan; sfyehe@tsmc.com (S.-F.Y.); mhwuzd@tsmc.com (M.-H.W.); kychouc@tsmc.com (K.-Y.C.); hytu@tsmc.com (H.T.); clleeza@tsmc.com (C.-L.L.); cyin@tsmc.com (C.Y.); 2 CEA, DAM, DIF, F-91297 Arpajon, France; philippe.paillet@cea.fr; 3 ISAE-SUPAERO, Université de Toulouse, 31055 Toulouse, France; vincent.goiffon@isae-supaero.fr

**Keywords:** CMOS image sensor (CIS), random telegraph signal (RTS), random telegraph noise (RTN), MOSFET channel RTN (MC-RTN), variable junction leakage (VJL), dark current (DC), gate induced drain leakage (GIDL), correlated double sampling (CDS), correlated multiple sampling (CMS), X-ray irradiation

## Abstract

In this paper we present a systematic approach to sort out different types of random telegraph noises (RTN) in CMOS image sensors (CIS) by examining their dependencies on the transfer gate off-voltage, the reset gate off-voltage, the photodiode integration time, and the sense node charge retention time. Besides the well-known source follower RTN, we have identified the RTN caused by varying photodiode dark current, transfer-gate and reset-gate induced sense node leakage. These four types of RTN and the dark signal shot noises dominate the noise distribution tails of CIS and non-CIS chips under test, either with or without X-ray irradiation. The effect of correlated multiple sampling (CMS) on noise reduction is studied and a theoretical model is developed to account for the measurement results.

## 1. Introduction

The readout random noise (RN) and the dark current (DC) are two key performance indices for CMOS image sensors (CIS). For state-of-art smartphones, CIS with 0.8 μm pixels have been in production in 2018–2019 [1,2,3,4,5]. The 0.7 μm [6] and 0.6 μm pixels are expected to come very soon. Given the small areas of these ever-shrinking pixels, the light sensitivities and the full-well capacities (FWC) are inevitably limited. Therefore, it is increasingly important to further drive down the RN and DC in order to achieve satisfactory signal-to-noise ratios. In literature and product datasheets, usually the average values of RN and DC are quoted. However, they are not sufficient to reveal the true performance of CIS, because the statistical distribution of RN and DC are typically non-Gaussian and highly asymmetrical with significant long tails. The values of RN and DC on the distribution tails could be 10 to 100 times higher than the average or the median of a large array.

The pixels with high DCs (white pixels) will cause pepper-and-salt fixed pattern noises (FPN), especially under low light conditions. The pixels with high RNs (blinking or twinkling pixels [7,8,9]) will create fixed-pattern temporal noises flickering from frame to frame. It is known that most of the pixels on the long distribution tails show the behavior of the random telegraph signals (RTS) [10,11,12,13,14,15,16,17,18,19,20,21,22,23]. Such noises are called the RTS noises, or the random telegraph noises (RTN). The pixels with RTN are referred to as the RTN pixels.

A general study of the RTS phenomena in semiconductor devices can be found in a recent book [24], covering a wide range of experimental and theoretical topics. For CIS, the most reported RTN originate from the source followers (SF) of the active pixels [10,11,12,13,14,15,16,17,18,19,20,21,22,23]. Although other sources, such as DC-RTS, have been reported [25,26], a comprehensive discrimination and comparison of the various sources of RTN in a CIS cannot be found in the literature yet.

The main objective of this paper is to highlight that there are many other RTN sources in CIS besides SF, in particular, the RTN caused by the varying photodiode dark current, the varying transfer-gate (TG), and reset-gate (RST) induced sense node (SN) leakage. In this work, various types of RN and RTN are analyzed systematically in both CIS and non-CIS chips, before and after X-ray irradiation. The RN/RTN composition among the noisiest pixels in a large array is studied.

The general effects of radiation damage on CIS and the design of radiation-hard CIS are outside the scope of this work [25,26,27,28,29,30,31,32]. Instead, X-ray irradiation is utilized as a tool to alter the composition of RTN types and to increase the number of RTN pixels for investigation. As reported in [20,21,22,23], the number of RTN pixels in an 8.3 MP CIS before X-ray irradiation is in the order of 1 percent. This RTN percentage can be increased significantly by X-ray irradiation. One unexpected finding during the course of this study is that the RTN due to the varying reset-gate induced SN leakage is identified in non-CIS test chips without any X-ray irradiation.

The rest of the paper is organized as follows. The test chips and their characteristics are presented in Section 2. A conceptual classification of different RTN types is given in Section 3. The effects of X-ray radiation damage on RN, DC, and the SN leakage are summarized in Section 4. The methods used to identify and to separate different RTN types in CIS, with and without X-ray irradiation, are discussed in Section 5. The identification of RTN due to the reset-gate induced SN leakage in a non-CIS chip is presented in Section 6. The effects of correlated multiple sampling (CMS) on RTN reduction are analyzed in Section 7. Finally, conclusions are drawn in Section 8. A mathematical model describing the time-dependent CMS is given in Appendix A.

Part of the results were published recently in [33,34].

## 2. Test Chip Design and Performance

**Figure 1:**
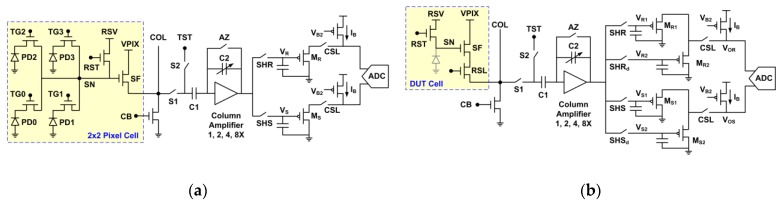
Analog signal chain schematic for (**a**) Chip-A and (**b**) Chip-B.

In this work, we study the random noises and leakage currents of two test chips. The first one, Chip-A, is an 8.3 MP, 1.1 μm pitch, stacked CIS. The top pixel array layer is fabricated in a 1P4M 45 nm Backside Illuminated (BSI) CIS process and the bottom ASIC layer is fabricated in a 1P6M 65 nm low-power mixed-mode process. Figure 1a shows the schematic of the analog signal chain. The pixel cell has a 2 × 2-shared structure without the row-select device. The column circuits between the pixel source follower (SF) and the global ADC are located on the bottom ASIC layer. The column amplifier provides an analog gain up to 8X and performs the correlated double sampling (CDS). The reset and signal voltages are sampled and held (S/H) on two capacitors, buffered by column PMOS source followers and digitized by external 14 bit ADCs at 10 Msps rates. The half-line even- and odd-column pixels are read out alternatively and reconstructed into a full line by an off-chip FPGA, which also generates the rolling shutter and other control signals. This chip achieves a low input-referred readout noise around 1.3 e-rms under an 8X gain [20,21,22,23].

**Figure 2:**
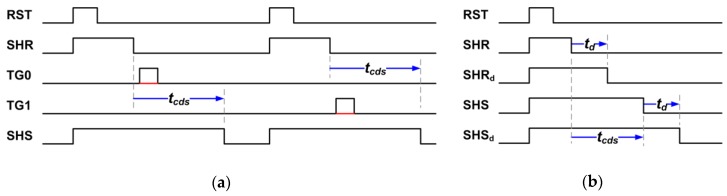
Simplified readout timing diagram for (**a**) Chip-A and (**b**) Chip-B.

A simplified timing diagram related to the CDS is illustrated in Figure 2a. The even- and odd-column pixels sharing the same column bus have to be read out sequentially, as indicated by the charge transfer signals TG0 and TG1. The time difference between the first S/H (SHR) and the second S/H (SHS) is denoted the CDS time ($t_{cds}$), or the sense-node charge retention time, which can be programmed from 0 to 25 μs. Note that although the reset KTC noises are eliminated by CDS, the noises of the SF or from the SN leakage cannot be cancelled by CDS.

The second chip, Chip-B, is a CIS-like test chip fabricated in a standard mixed-mode, non-CIS, 1P6M, 40 nm, low-power process. The DUT cell structure is similar to a 3-transistor (3T) pixel with no transfer gate. There is no intentional photodiode (PD), but the source diffusion region of the reset NMOS (RST) and the substrate forms a parasitic diode, grey-colored in Figure 1b schematic. The column circuits are similar to those of Chip-A, except that each of the S/H circuit is split into 2 branches in Chip-B for the purpose of correlated multiple sampling (CMS). Even without the photodiodes, Chip-B allows us to characterize the dark random noise and the sense node (SN) leakage similar to a real CIS.

The simplified readout timing diagram for Chip-B is given in Figure 2b. In contrast to Chip-A, Chip-B samples the reset (signal) voltage twice (i.e., CMS) as indicated by SHR and SHR_d_ (SHS and SHS_d_), separated by a delay time ($t_{d}$) programmable from 0 to 11.5 μs. The CDS time ($t_{cds})$ can be independently programmable up to 25 μs while the condition $t_{d}<t_{cds}$ is always satisfied. It should be noted that Chip-B is intentionally operated with a timing sequence similar to a 4T pixel, rather than a 3T pixel, as shown in Figure 2b. Table 1 lists the key parameters and operation voltages for both Chip-A and Chip-B, both running at approximate rates of 1 frame per second.

## 3. Different RTN Types and Sources

It is known in literature that there are generally two kinds of RTN [24]. One is the MOSFET channel RTN (MC-RTN); the other is the RTN caused by the variable junction leakage (VJL) [24]. The MC-RTN is typically attributed to the capture and emission of majority carriers by traps near the Si-SiO_2_ interface or inside the gate oxide bulk. The random trapping and de-trapping process causes an equivalent fluctuation in threshold voltage ($V_{th}$). The RTN from the SF $V_{th}$ fluctuation (SF-RTN) belongs to the category of MC-RTN. The VJL related RTN refers to the noises generated by the random switching of a junction leakage between two or multiple levels. Such a junction is typically connected to a MOSFET switch in the OFF state. Therefore, it is difficult to separate the junction leakage from the MOSFET off-current and the gate-induced drain leakage (GIDL). The physical mechanism of the VJL is typically described as a trap-assisted tunneling (TAT) or a meta-stable atomic configuration of a trap causing the Shockley–Read–Hall (SRH) recombination process [35,36,37,38,39,40,41].

**Figure 3:**
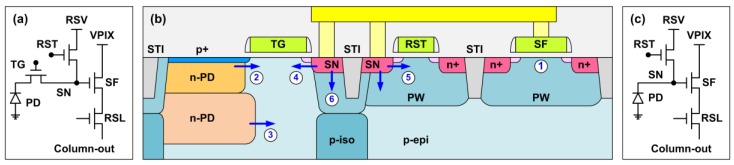
(**a**) A 4T pinned photodiode pixel in Chip-A, (**b**) a simplified cross section for the 4T pixel, (**c**) a 3T pixel in Chip-B. The 6 RTN types in Table 2 are labelled accordingly in (**b**).

With the above concepts, we can envision 6 RTN types in an active pixel according to the physical locations (SF, PD, or SN) and the mechanisms (gate-induced or not gate-induced), listed in Table 1. The active pixel with a pinned photodiode (PPD) and a transfer gate (TG) is commonly called a 4-transistor (4T) pixel for convenience, although the average number of transistors per pixel could be 3, 2.5, 2, 1.75, 1.5, 1.375, or 1.25, depending on whether there is a row-select device (RSL) and whether it is a 1 × 1, 1 × 2, 2 × 2, or 2 × 4-shared structure. In Table 2, the 3-transistor (3T) active pixel with no TG is included for comparison. These six RTN sources are illustrated in a simplified pixel cross section, together with the schematics of the 4T and 3T pixels, in Figure 3.

## 4. Effects of X-Ray Radiation Damage

**Figure 4:**
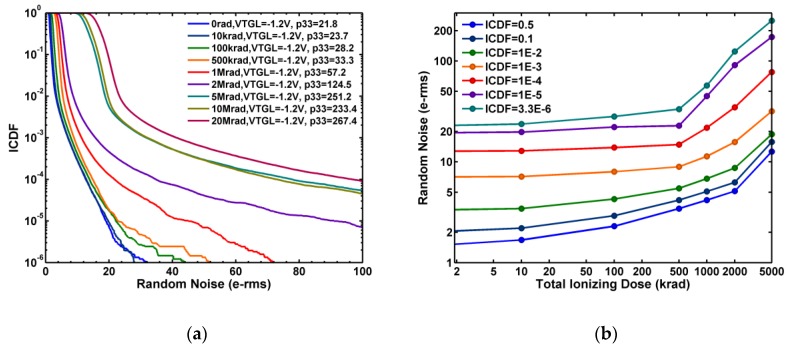
(**a**) Statistical distribution (ICDF) of the random noises (RN) indexed by the total ionizing dose (TID) of X-ray from 0 to 20 Mrad (SiO_2_), where p33 denotes the RN at ICDF = 3.3 ppm. (**b**) RN as a function of TID at several constant ICDF values from 3.3 ppm to 0.5.

It is generally known that ionizing radiation can degrade the performance of CIS in terms of increasing DC, SN leakage, RN, and RTN [25,26,27,28,29,30,31,32]. To study the radiation damage effects, several Chip-A samples are irradiated, while grounded, by 10 keV X-ray at CEA-DAM facility at room temperature, with 0 rad, 10 krad, 100 krad, 500 krad, 1 Mrad, 2 Mrad, 5 Mrad, 10 Mrad, and 20 Mrad (SiO_2_) total ionizing dose (TID), respectively [23]. All data below were measured at room temperature. Figure 4a shows a systematic degradation of RN inverse cumulative distribution functions (ICDF) as the TID is increased. It can be seen that both the flicker noises dominated region near ICDF = 0.5 (median) and RTN dominated region with ICDF < 0.001 (the long tails) are shifted towards higher values at higher TID. The constant ICDF contours plotted in Figure 4b indicate that the degradation is accelerated in the higher TID regions.

It is found that up to 1 Mrad, the readout circuits show negligible performance degradation, verified under a test mode bypassing the pixel array [23]. The circuit-only RN histograms are close to ideal Gaussian shapes with no noticeable long tails [23]. The median circuit noises are in the range of 0.5−0.7 e-rms, much smaller than the pixels and circuit combined noises around 1.3 e-rms [23]. Therefore, it can be concluded that the RN and RTN degradation induced by X-ray mainly comes from the pixels, not the readout circuits.

**Figure 5:**
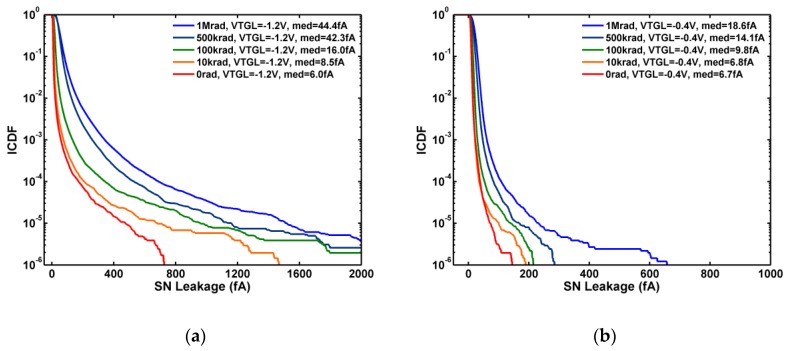
Statistical distribution of the SN leakage indexed by TID. (**a**) Under the default bias, VTGL = −1.2 V; (**b**) under VTGL = −0.4 V, the GIDL-RTN is significantly reduced.

Both the sense node (SN) leakage and photodiode dark current (DC) are systematically increased as TID increases [23]. A notable feature of the SN leakage statistics is that the extensive long tails generated by X-ray irradiation can be suppressed by raising the TG off-voltage (VTGL) from the default −1.2 V to −0.4 V as compared in Figure 5a,b. This is considered as an indirect evidence to link the TG gate-induced drain leakage (GIDL) to increased RTN due to X-ray irradiation. More direct evidence will be presented in the next section.

## 5. Identification of SF-RTN, DC-RTN, and Transfer-Gate GIDL-RTN in Chip-A

The RN statistical distributions, often presented in histograms or ICDF curves, do not provide information about the nature of the RTN. To identify the different types of RTN, we have to inspect individual pixel’s dark signal waveforms and their dependences on the PD integration time, the SN charge retention time, the SN voltage, and the gate bias of the transfer- and reset-transistors. In this study, we examine the noisiest 4000 pixels of the 8.3 MP CIS, with or without X-ray irradiation, under various bias voltages and integration times. Four types of RN have been clearly identified: the SF-RTN, the DC-RTN, the TG GIDL-RTN, as well as the dark signal shot noises. The criteria of sorting the RN into 4 types are based on their behaviors summarized in Table 3 below.

**Figure 6:**
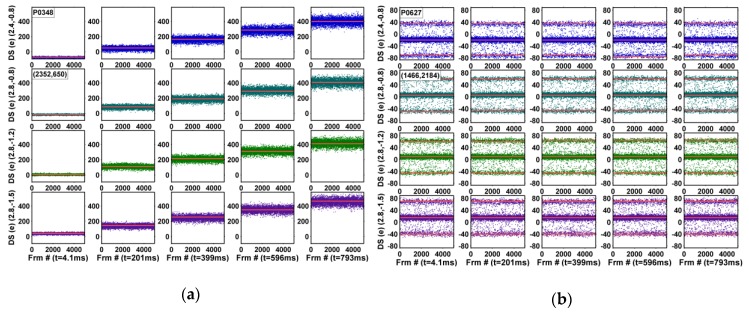
(**a**) An example of dark signal shot noise; (**b**) an example of a SF-RTN pixel. In Figure 6, Figure 7 and Figure 8 5000-frame dark signal (DS) 5 × 4 combo plots, the unique pixel number and its (column, row) coordinates are labeled on the top-left. The 5 integration times, from 4.1 ms to 793 ms, are labeled along the horizontal directions and the 4 operation voltages, (RSVH, VTGL) = (2.4 V, −0.8 V), (2.8 V, −0.8 V), (2.8 V, −1.2 V), and (2.8 V, −1.5 V), are labeled along the vertical directions, where RSVH is the on-voltage of RSV and VTGL is the off-voltage of TG.

**Figure 7:**
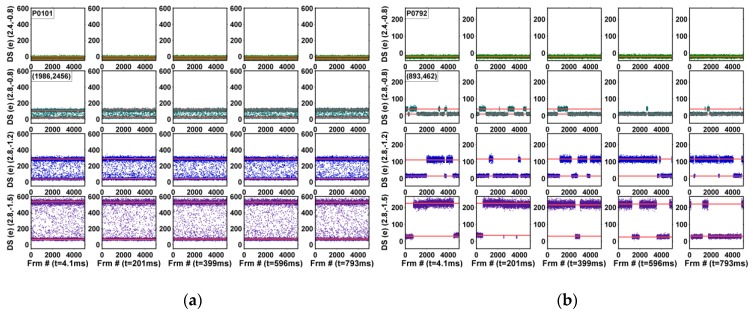
Examples of GIDL-RTN pixels with (**a**) short time constants, and (**b**) long time constants.

**Figure 8:**
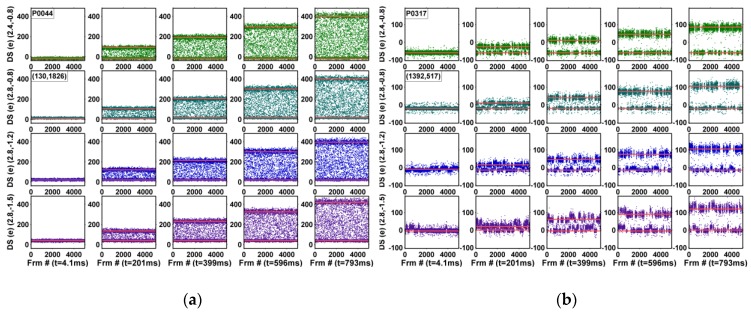
Examples of DC-RTN pixels with (**a**) short time constants, and (**b**) long time constants.

The input-referred dark signals, in the units of electrons, are measured under an 8X gain and the normal TG operation. In comparison, the study of the dependency on SN charge retention time reported in [23] was measured under a special test mode with the TG turned off during the CDS operation such that the DC would not mix up with the SN leakage. The noise waveforms of 5000 consecutive frames for all 4000 pixels are plotted in 5 × 4 composite graphs; the 5 integration times, from 4.1 ms to 793 ms, are along the horizontal directions and the 4 operation voltages, (RSVH, VTGL) = (2.4 V, −0.8 V), (2.8 V, −0.8 V), (2.8 V, −1.2 V), and (2.8 V, −1.5 V), are along the vertical directions, where RSVH is the on-voltage of RSV and VTGL is the off-voltage of TG. The corresponding TG drain-to-gate voltages ($V_{DG}$) are 2.9 V, 3.3 V, 3.7 V, and 4.0 V, approximately. Six example pixels are shown in Figure 6, Figure 7 and Figure 8 and discussed below.

Figure 6a shows a typical pixel with high dark signal (DS) shot noises. The dark signal is linearly proportional to the integration time, but insensitive to the (RSVH, VTGL) voltages. The shot noise, calculated as the RMS of the dark signal, is proportional to the square root of the signal average, following the well-known Poisson statistics. Figure 6b shows a sample SF-RTN pixel with 3 symmetrically centered discrete levels due to the effect of CDS [20,21,22,23]. The amplitude of the SF-RTN shows a very weak or no dependence on either the integration time or the operation voltages. The time constants of the SF-RTN are typically shorter than the CDS time such that the RTN trap may randomly switch between 2 states between the first and the second sampling to generate the observed 3 levels.

Figure 7 shows 2 representative pixels with GIDL-RTN. The RTN amplitudes are independent of the integration time, but are strongly dependent on the operation voltage. The RTN are highest under (RSVH, VTGL) = (2.8 V, −1.5 V) and are suppressed dramatically under (2.4 V, −0.8 V). The GIDL-RTN is caused by the random switching of the SN leakage between 2 discrete values. The time constants of the switching are typically much longer than the CDS time such that the leakage remains unchanged between the first and the second sampling, resulting in only 2 observable signal levels, unlike the 3-level SF-RTN. The pixel in Figure 7a shows a time constant comparable to, or shorter than, the frame time (about 1 sec). The pixel in Figure 7b shows a time constant considerably longer than the frame time; the signal could get stuck at one state up to hours before switching to the other state.

Figure 8 shows 2 selected pixels with DC-RTN; the RTN amplitudes are linearly proportional to the PD integration time, but independent of the operation voltages. Figure 8a is one example with a time constant comparable to or shorter than the frame time (but still longer than the CDS time); Figure 8b is one example with a time constant much longer than the frame time.

**Figure 9:**
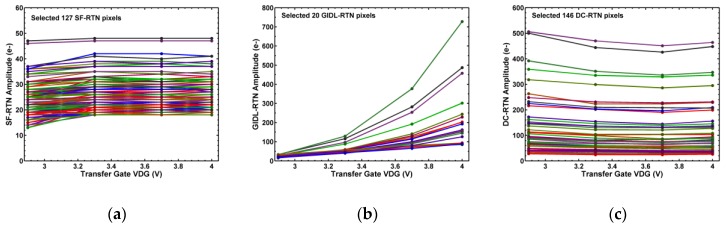
Dependence of RTN amplitude (integration time = 793 ms) on transfer-gate $V_{DG}$ for a number of selected pixels: (**a**) SF-RTN, (**b**) GIDL-RTN, and (**c**) DC-RTN.

The dependence of RTN amplitude on the TG drain-to-gate voltage ($V_{DG}$) is compared in Figure 9, further highlighting the different behaviors of various RTN types. It is evident that the GIDL-RTN is highly enhanced by increasing $V_{DG}$, while the SF-RTN and DC-RTN are almost independent of $V_{DG}$.

**Figure 10:**
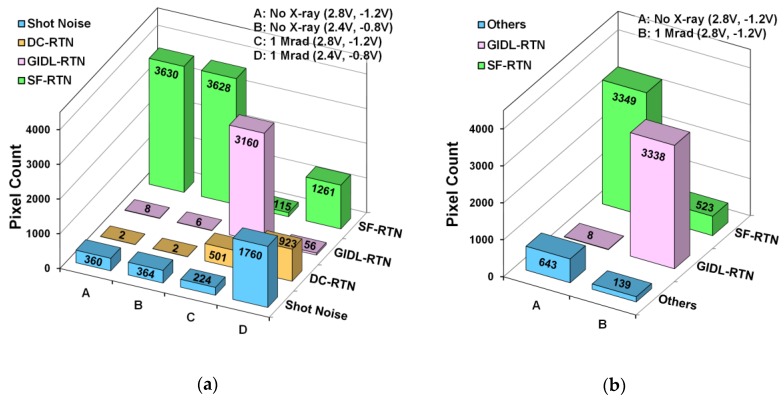
Composition of various noise types of 4000 noisiest pixels before and after X-ray irradiation; (**a**) TG pulses enabled during CDS; (**b**) TG pulses disabled during CDS [23]. The operation voltages (RSVH, VTGL) are shown in the legend.

The sorting of the noisiest 4000 pixels is performed semi-automatically, first by a MATLAB program; then double-checked and corrected by visually inspecting the DS waveforms. The results are compared in Figure 10a for 2 chips, one without X-ray irradiation and one irradiated by 1 Mrad X-ray, each measured under 2 bias conditions, (RSVH, VTGL) = (2.8 V, −1.2 V) and (2.4 V, −0.8 V), respectively. The (2.8 V, −1.2 V) is the standard (default) bias condition; the (2.4 V, −0.8 V) is the GIDL-reduced bias condition. Before X-ray irradiation, the dominant RTN type is clearly the SF-RTN, regardless of the standard or the GIDL-reduced biases. Very few pixels show DC-RTN or GIDL-RTN. In contrast, a dramatic increase of DS shot noise, DC-RTN, and GIDL-RTN is observed in the 1 Mrad chip. Under the standard bias (RSVH, VTGL) = (2.8 V, −1.2 V), the GIDL-RTN dominates; while under the GIDL-reduced bias, (RSVH, VTGL) = (2.4 V, −0.8 V), the GIDL-RTN is significantly suppressed to almost zero while the shot noise, SF-RTN, and DC-RTN together become dominant.

The data in Figure 10a are measured with the TG pulses enabled during the CDS (normal operation). For comparison, the data reported in [23] are measured with the TG pulses disabled during the CDS (test mode). They are plotted in Figure 10b, where the DC-RTN and DS shot noises not observable without the charge transfer from PD to SN. The common feature of Figure 10a,b is that the SF-RTN dominates in the chip without X-ray and the GIDL-RTN dominates in the chip after 1 Mrad X-ray irradiation.

**Figure 11:**
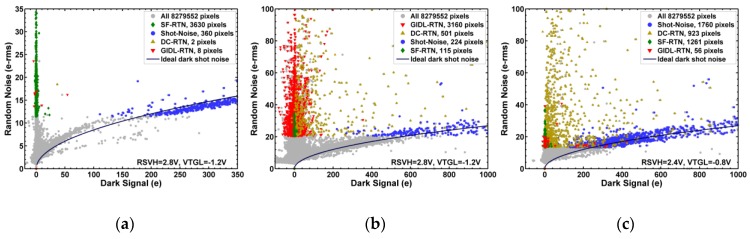
Correlation between random noise (RN) and dark signal (DS). The ideal shot noise curve is described by the equation $RN\;\sim \;\sqrt{DS}$. The 3 cases being compared are: (**a**) no X-ray irradiation, RSVH = 2.8 V, VTGL = −1.2 V; (**b**) 1 Mrad X-ray, RSVH = 2.8 V, VTGL = −1.2 V; (**c**) 1 Mrad X-ray, RSVH = 2.4 V, VTGL = −0.8 V.

**Figure 12:**
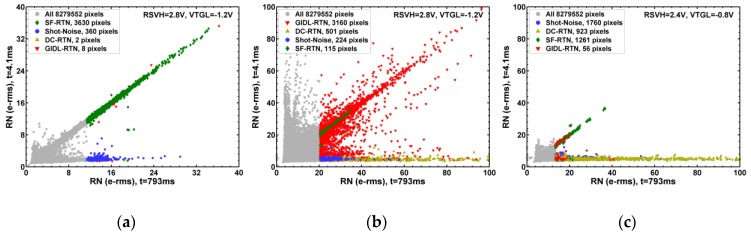
Correlation between RN measured with short (4.1 ms) integration time vs. RN measured with long (793 ms) integration time. The 3 cases being compared are: (**a**) no X-ray irradiation, RSVH = 2.8 V, VTGL = −1.2 V; (**b**) 1 Mrad X-ray, RSVH = 2.8 V, VTGL = −1.2 V; (**c**) 1 Mrad X-ray, RSVH = 2.4 V, VTGL = −0.8 V.

**Figure 13:**
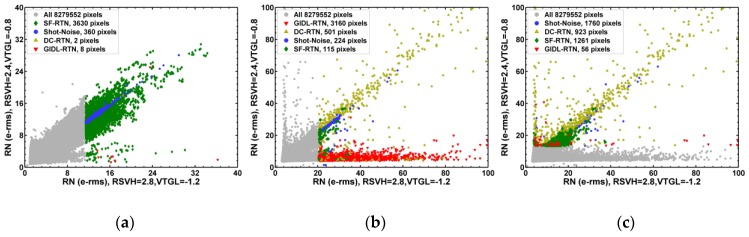
Correlation between RN measured with the standard bias vs. the GIDL-reduced bias. The 3 cases being compared are: (**a**) no X-ray irradiation, RSVH = 2.8 V, VTGL = −1.2 V; (**b**) 1 Mrad X-ray, RSVH = 2.8 V, VTGL = −1.2 V; (**c**) 1 Mrad X-ray, RSVH = 2.4 V, VTGL = −0.8 V.

The different behaviors of the 4 types of noises can be further illustrated in Figure 11, Figure 12 and Figure 13 correlation plots. The sorted RN data points are represented by different marker shapes and colors; the full 8.3 MP data are included on the background. From Figure 10a, we can see that the bias voltages have no observable impact on the chip without X-ray. Therefore, in Figure 11, Figure 12 and Figure 13 we will focus on the comparison of the 3 cases: (a) no X-ray irradiation, RSVH = 2.8 V, VTGL = −1.2 V; (b) 1 Mrad X-ray, RSVH = 2.8 V, VTGL = −1.2 V; (c) 1 Mrad X-ray, RSVH = 2.4 V, VTGL = −0.8 V.

Figure 11a–c are the RN versus DS scatter plots. For the chip with no X-ray in Figure 11a, the SF-RTN pixels concentrated in the region of high RN but low DS, clearly separated from the shot-noise pixels in the region of relatively low RN but high DS. Furthermore, the relationship between RN and DS for the shot-noise pixels follows the ideal Poisson statistics $RN\;\sim \;\sqrt{DS}$ very well. For the 1 Mrad chip under standard bias condition in Figure 11b, the GIDL-RTN becomes dominant in the region of high RN but low DS. For the 1 Mrad chip under the GIDL-reduced bias condition in Figure 11c, the GIDL-RTN is essentially suppressed. It is interesting to point out that the pixels with high dark signals are not necessarily the pixels showing DC-RTN, vice versa. The DC-RTN pixels and the DS shot-noise pixels are not mixed at all in the correlation plot.

Figure 12 shows the correlation of RN with short PD integration time vs. RN with long integration time. In Figure 12a, the SF-RTN and the DS shot-noise pixels are clearly decoupled into 2 branches. The SF-RTN pixels are narrowly distributed along the diagonal line, showing no dependence on integration time; while the DS shot-noise pixels depend on integration time. The GIDL-RTN pixels in Figure 12b are basically along the diagonal direction, but widely spreading out. The DC-RTN pixels in Figure 12c show higher RN and stronger dependence on integration time than the DS shot-noise pixels in Figure 12a. Figure 13 shows the correlation between RN measured with the standard bias vs. RN measured with the GIDL-reduced bias. The data in Figure 13b,c are the same, but the 2 plots show that the 4000 noisiest pixels selected under one bias condition are totally different from those selected under a different bias condition.

The main point is that when RN is viewed merely as a data point in histograms or ICDF curves, the details about the nature of the RN are lost. However, when the RN of individual pixel is studied through its time-domain waveform, its dependence on bias condition, integration time, and SN charge retention time [23], it becomes very clear that different types of RN can be unambiguously identified and distinguished.

## 6. Identification of Reset-Gate GIDL-RTN in Chip-B

In this section, we switch the subject to the non-CIS Chip-B (standard 40 nm process) operated under the CDS mode with the time delay $t_{d}$ set to zero. The effects of non-zero $t_{d}$ (the CMS mode) is discussed in the Section 7.

**Figure 14:**
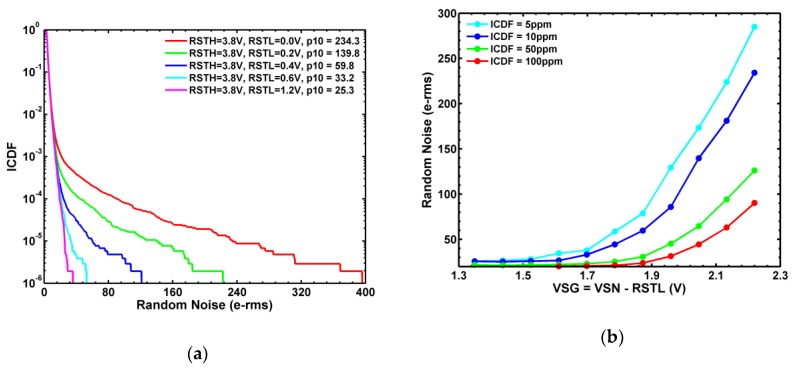
(**a**) A family of RN ICDF curves indexed by varying RSTL, where p10 denotes the RN values at ICDF = 10 ppm; (**b**) the RN constant ICDF contours versus V_SG_ of the RST transistor.

Figure 14a shows the ICDF plot of the RN under CDS time = 25 μs, RSVH = 2.8 V, RSTH = 3.8 V, and various RST off-voltage voltages (RSTL). First, we notice that the RN tail can be gradually suppressed by raising the RSTL from the default 0 V towards 1.2 V. This behavior is strikingly similar to the suppression of the RN tail by raising the VTGL of Chip-A with 1 Mrad X-ray irradiation [23]. Especially, the exponential dependence of RN on the RST source-to-gate voltage ($V_{SG}$) in Figure 14b is similar to the dependence on $V_{DG}$ of TG in Figure 9b. Based on this similarity, we speculate that the RTN tail is caused by the RST gate induced SN leakage.

**Figure 15:**
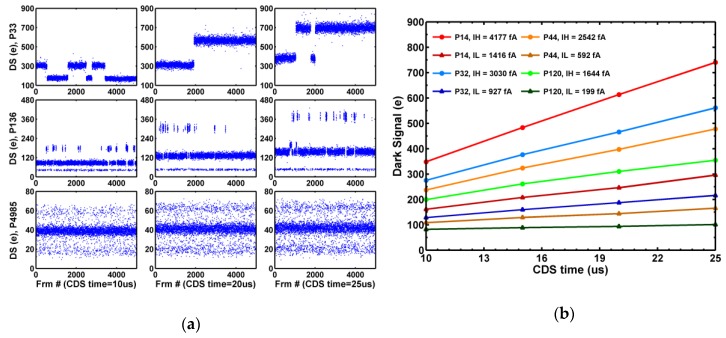
(**a**) Dark signal waveforms of 5000 frames for 3 selected pixels measured at CDS time = 10 μs, 20 μs, and 25μs. The top row shows a GIDL-RTN pixel with 2 discrete states; the middle row shows a GIDL-RTN pixel with 3 discrete states, and the bottom row shows a SF-RTN pixel. (**b**) Dark signal vs. CDS time for 4 selected GIDL-RTN pixels with 2 discrete states, where IH and IL are the high and low SN leakage.

Similar to the TG GIDL-RTN, the RST GIDL-RTN can also be identified by inspecting the individual pixel noise waveforms and its dependence on the SN charge retention time (CDS time). Figure 15a shows 3 example pixels. The top 2 pixels have RTN amplitudes linearly proportional to CDS time; therefore, are designated as the GIDL-RTN pixels. The bottom pixel shows symmetric 3 discrete levels and no dependence on CDS time; therefore, is classified as a SF-RTN pixel. The signal levels of the high and low leakage states are plotted in Figure 15b for 4 selected GIDL-RTN pixels with 2 discrete leakage levels (IH and IL) as functions of CDS time. The measured SN leakage currents of the 1M array DUTs vary widely from below fA to above pA.

**Figure 16:**
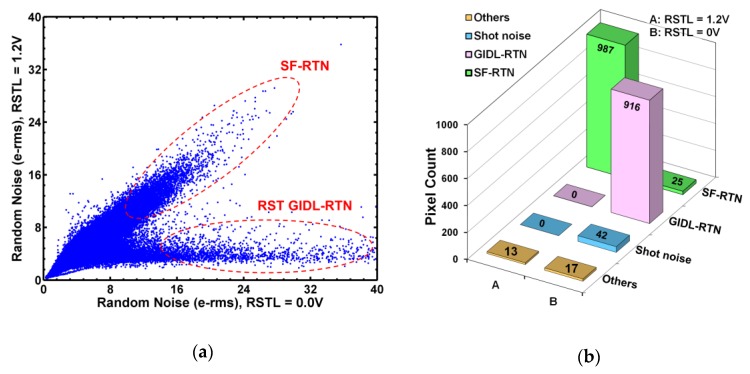
(**a**) The correlation between RN at RSTL = 1.2 V and RN at RSTL = 0 V for all 1.04 × 10^6^ pixels. (**b**) The results of sorting the 1000 noisiest pixels into 4 different groups.

A correlation plot in Figure 16a between RN at RSTL = 1.2 V and RN at RSTL = 0 V shows that the population is apparently split into 2 branches. The SF-RTN pixels along the diagonal line are independent of RSTL. The RST GIDL-RTN pixels on the lower branch are strongly dependent on RSTL. This is again similar to the dependence of TG GIDL-RTN pixels on VTGL shown in Figure 10 of [23]. Using the same methodology described in Section 5, the results of sorting the 1000 noisiest pixels are given in Figure 16b, based on a reduced set of criteria in Table 4 below. It is evident that the dominant RTN type is the GIDL-RTN at RSTL = 0 V, but SF-RTN becomes dominant at RSTL = 1.2 V when the GIDL-RTN is suppressed. Almost no DC-RTN pixels are detected.

It is interesting to highlight the observed differences between the CIS Chip-A in Section 5 and the non-CIS Chip-B in Section 6. For Chip-A, the number of GIDL-RTN pixels is negligible in the sample without X-ray irradiation for both of the TG GIDL-RTN and the RST GIDL-RTN. The TG GIDL-RTN is found dominant in the sample irradiated by 1 Mrad X-ray, but there is no evidence of any RST GIDL-RTN at RSTL = 0 V. In contrast, the pixels with RST GIDL-RTN are abundant in Chip-B even without X-ray irradiation at RSTL = 0 V. We suspect that the differences are due to the threshold voltages, the device dimensions, and the device designs of two RST transistors in Chip-A (customized CIS) and Chip-B (standard non-CIS). The detailed study is a work in progress and may be published elsewhere in the future.

## 7. The Effects of Multiple Sampling

**Figure 17:**
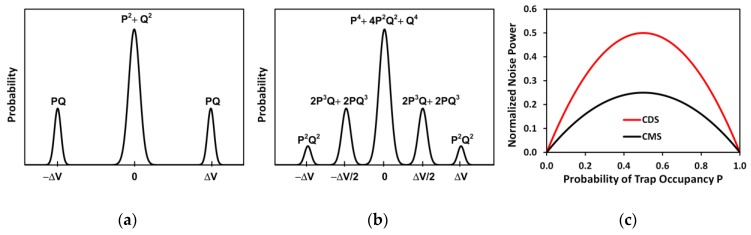
(**a**) A hypothetical signal histogram as a result of CDS, P is the trap occupancy probability and Q = 1 − P; (**b**) the signal histogram as a result of CMS; (**c**) the calculated noise power for CDS and CMS normalized to ${\left(\Delta V\right)}^{2}$ as functions of probability P.

For Chip-A described in Figure 1a and Figure 2a, the voltages before and after the charge transfer are sampled by SHR and SHS signals for CDS operation. On the other hand, the Chip-B in Figure 1b and Figure 2b is designed to implement the correlated multiple sampling (CMS) for the purpose of noise reduction [42,43,44,45,46,47,48,49]. The reset and signal voltages are sampled twice by the pairs of pulses (SHR, SHR_d_) and (SHS, SHS_d_), respectively. The time delay ($t_{d}$) between the falling edge of SHR_d_ (SHS_d_) and the falling edge of SHR (SHS) is programmable from 0 to 11.5 μs. In this design, the source terminals of two PMOS source followers in the column buffers are shorted together and sourced by a current $I_{B}$, same as in Chip-A. The output voltages $V_{OR}$ and $V_{OS}$ can be calculated from the sampled $V_{R1}$, $V_{R2}$, $V_{S1}$, and $V_{S2}$ as:

(1) $$V_{OR}=\frac{1}{2}\left(V_{R1}+V_{R2}\right)+V_{tp}+\frac{1}{2}\sqrt{-{\left(V_{R1}-V_{R2}\right)}^{2}+4I_{B}\left(L/W\right)/\left(\mu_{p}C_{ox}\right)}\;\sim \;\frac{1}{2}\left(V_{R1}+V_{R2}\right),$$

(2) $$V_{OS}=\frac{1}{2}\left(V_{S1}+V_{S2}\right)+V_{tp}+\frac{1}{2}\sqrt{-{\left(V_{S1}-V_{S2}\right)}^{2}+4I_{B}\left(L/W\right)/\left(\mu_{p}C_{ox}\right)}\;\sim \;\frac{1}{2}\left(V_{S1}+V_{S2}\right),$$

where $\mu_{p}$ is the hole mobility, $C_{ox}$ is the gate oxide capacitance, $V_{tp}$ is the PMOS source follower threshold voltage, and (L/W) is the length/width. Since $V_{R1}$ and $V_{R2}$ are sampled from the same source, they are roughly equal. Under this approximation, $V_{OR}\;\left(V_{OS}\right)$ can be simplified to the average of $V_{R1}$ and $V_{R2}$ ($V_{S1}$ and $V_{S2}$), respectively. The differential output $V_{OR}-V_{OS}$ is digitized by the ADC, similar to the CDS operation. The benefit of averaging two sampled voltages is that the uncorrelated noises from the pixel SF, which is not cancelled by CDS, will be reduced by a factor of $\sqrt{2}$ in CMS. Furthermore, the SF-RTN is reduced at the same time. This can be understood using a simple model in Figure 17. Suppose the SF-RTN is due to a single trap with 2 discrete levels differing by ΔV (the RTN amplitude) and the probability of trap occupancy (PTO) is P. In the case of $t_{d}=0$, the result of CDS subtraction would generate a noise histogram with 3 discrete peaks at −ΔV, 0, and ΔV, referenced to the mean value, as illustrated in Figure 17a. However, for the CMS operation with $t_{d}\neq 0$, the 4 samplings ($V_{R1},\;V_{R2},\;V_{S1},\;V_{S2}$) and the approximate Equations (1) and (2) lead to a noise histogram in Figure 17b with 5 discrete levels. The probabilities labeled in the graphs are calculated by assuming the 4 samplings are statistically independent. The noise power normalized to ${\left(\Delta V\right)}^{2}$ can be calculated and plotted in Figure 17c, showing an approximate factor-of-2 reduction from CDS to CMS.

**Figure 18:**
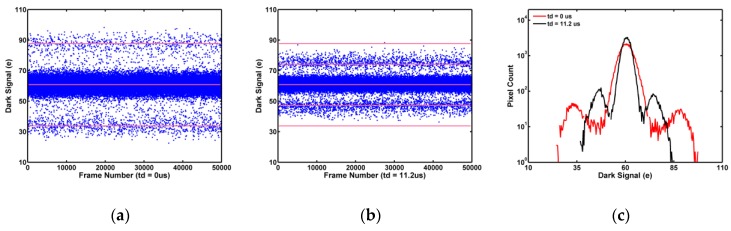
Dependence of SF-RTN waveforms on time delay $t_{d}$; (**a**) $t_{d}=0$, CDS; (**b**) $t_{d}=$ 11.2 μs, CMS; (**c**) comparison of CDS and CMS histograms.

The measured data for one selected pixel with SF-RTN is shown in Figure 18a with $t_{d}=0$ (CDS), and in Figure 18b $t_{d}=$ 11.2 μs (CMS). The two histograms are compared in Figure 18c. Although we are not able to find a better example showing an exact transition from the 3-peak CDS histogram to the 5-peak CMS histogram predicted by the simple model, the shift of the probability peaks from the locations at ±ΔV to the locations at ±ΔV/2 and the reduction of RTN noise power are nevertheless well verified. The reason of the discrepancy could be that the simple model does not account for the effects of finite circuit settling time, the non-RTN (flicker and thermal) noises, and the approximations made in deriving Equations (1) and (2).

**Figure 19:**
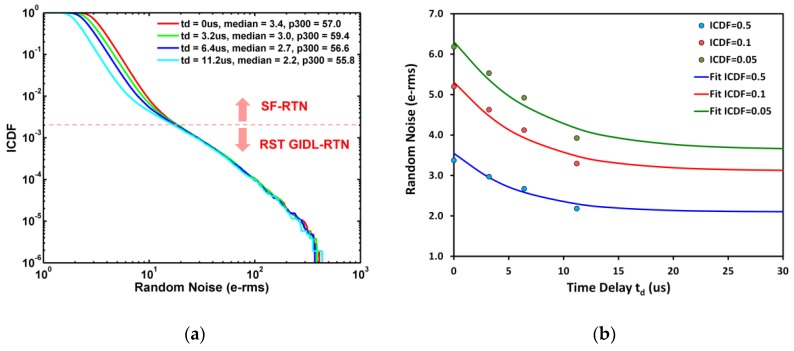
(**a**) Random noises ICDF curves indexed by time delay ($t_{d}$) where p300 denotes the RN at ICDF = 300 ppm and the red dash line corresponds to ICDF = 0.002; (**b**) RN at ICDF = 0.5 (median), 0.1, and 0.05 as functions of $t_{d}$.

In addition, we can calculate the RTN noise power reduction as a function of $t_{d}$ using this model. The results in Figure 17 are based on an implicit assumption that both $t_{cds}$ and $t_{d}$ are much longer than the characteristic time constant τ of the RTN trap. A more general formula with $t_{d}$-dependence is derived in Appendix A. A family of measured RN distributions indexed by $t_{d}$ is shown in Figure 19a. It can be seen that the ICDF curves below 0.002 are independent of $t_{d}$, because the GIDL-RTN dominates in this regime. The GIDL-RTN typically has a time constant much longer than the CDS/CMS time such that the GIDL leakage remains unchanged during the CDS/CMS operation. On the contrary, in the regime with ICDF above 0.002, the SF-RTN dominates and the time constant is typically shorter than the CDS/CMS time. Therefore, a noise reduction from CDS ($t_{d}=0$) to CMS ($t_{d}=11.2$ μs) in the SF-RTN dominated regime (ICDF > 0.002) matches the model prediction, Equation (A12), reasonably well, as shown in Figure 19b. In other words, the CMS noise reduction is only effective for SF-RTN with shorter time constants, not for GIDL-RTN with longer time constants.

## 8. Conclusions

Continued improvement of RTN is essential for enhancing CIS performance when the pixel scales down to 0.7 μm pitch and beyond. Understanding the RTN behavior and classification of the RTN pixels into different types are the necessary first step in order to reduce RTN through pixel design and minimizing process-induced damage (PID). In this paper, we identified the SF-RTN, the DC-RTN, the TG GIDL-RTN, and the RST GIDL-RTN in active pixels according to their dependence on the PD integration time, the SN charge retention time, the $V_{DG}$ across the TG device, and the $V_{SG}$ across the RST device, in CIS and non-CIS chips, with and without X-ray irradiation. For instance, in noise reduction multiple-split experiments, the RTN classification methodology presented in Section 5 can be used effectively to identify which RTN type is affected by which process split.

We further studied the effect of CMS as a useful technique for RTN reduction through circuit design. A theoretical model was presented to account for the time-dependence of the effectiveness of CMS, which explained the measured data reasonably well.

The process nodes used to manufacture the pixel-array and the ASIC layers in stacked CIS are expected to move down the path of the Moore’s Law gradually. Extending the study of RTN to high-K metal gate and FinFET technologies is an important goal for our future investigation.

## Figures and Tables

**Table 1:** Characteristics of Chip-A, Chip-B, and operation voltages.

Parameters	Chip-A	Chip-B	Operation Voltage		Chip-A	Chip-B
Cell size (um^2^)	1.1 × 1.1	3 × 2.6	RST	RSTH	3.1 V	3.8 V
Array size	3296 × 2512	1300 × 800		RSTL	0.0 V	0.0 V
SF W/L (um/um)	0.2/0.8	0.9/1.8	RSV	RSVH	2.8 V	2.8 V
RST W/L (um/um)	0.2/0.4	0.9/0.79		RSVL	0.7 V	N/A
SN capacitance	1.34 fF	3.3 fF	TG	VTGH	2.8 V	N/A
SN conversion gain	119 uV/e−	48.5 uV/e−		VTGL	−1.2 V	N/A
RN at 8X gain	1.3 e−	3.4 e−	VPIX	fixed	2.7 V	2.7 V
(**a**)			(**b**)			

**Table 2:** Conceptual RTN types in active pixels.

Location	Mechanism	4T	3T	Nickname
SF	① SF MOSFET channel RTN	Yes ^†^	Yes ^‡^	SF-RTN
PD	② Transfer-gate-induced PD leakage	Yes *	N/A	N/A
	③ Non-gate-induced PD leakage	Yes ^†^	Yes *	DC-RTN
SN	④ Transfer-gate induced SN leakage	Yes ^†^	N/A	GIDL-RTN
	⑤ Reset-gate induced SN leakage	Yes *	Yes ^‡^	GIDL-RTN
	⑥ Non-gate-induced SN leakage	Yes *	Yes *	N/A

^†^ Identified in Chip-A. ^‡^ Identified in Chip-B. * Not observed in this work.

**Table 3:** Five types of random noises and their dependencies on voltage and time in Chip-A.

Random Noise Behavior	SF-RTN	TG ^†^ GIDL-RTN	RST ^‡^ GIDL-RTN	DC-RTN	Shot Noises
RTS behavior	Yes	Yes	Yes	Yes	No
PD integration time	No	No	No	Yes	Yes
RSVH & VTGL voltages	No *	Yes	No	No	No
RSVH & RSTL voltages	No *	No	Yes	No	No
SN charge retention time	No	Yes	Yes	No	No

The voltage names RSVH, RSTL, and VTGL are defined in Table 1. * Very weak dependence in the range of the experiment. ^†^ For Chip-A, only the TG GIDL-RTN is observed, but not the RST GIDL-RTN. ^‡^ RST GIDL-RTN is observed in Chip-B, to be discussed in Section 6.

**Table 4:** Four types of random noises and their dependencies on voltage and time in Chip-B

Random Noise Behavior	SF-RTN	RST GIDL-RTN	DC-RTN	Shot Noise
RTS behavior	Yes	Yes	Yes	No
RSVH and RSTL voltages	No	Yes	No	No
SN charge retention time ^†^	No	Yes	Yes	No

^†^ There is no TG in Chip-B pixel; the parasitic PD is connected to SN directly; therefore, the PD integration time is the same as the SN charge retention time (in this case, the CDS time: $t_{cds}$).

## Appendix A: Time-Dependent RTN Model for Correlated Multiple Sampling

Consider a single-carrier trap with 2 discrete states; either occupied or empty. Assume that the time-dependent probability of trap occupancy P(t) is governed by the first-order differential equation:

(A1) $$\frac{dP\left(t\right)}{dt}=-\frac{P\left(t\right)}{\tau_{e}}+\frac{1-P\left(t\right)}{\tau_{c}},$$

where $\tau_{e}$ is the emission time constant and $\tau_{c}$ is the capture time constant of the trap. The general solution of Equation (A1) is:

(A2) $$P\left(t\right)=P+\left(P\left(0\right)-P\right)e^{-t/\tau },$$

where $\tau ≝\tau_{e}\tau_{c}/\left(\tau_{e}+\tau_{c}\right)$ is defined as the system characteristic time constant; $P≝\tau_{e}/\left(\tau_{e}+\tau_{c}\right)$ and $Q≝\tau_{c}/\left(\tau_{e}+\tau_{c}\right)$ are probabilities of being occupied or empty at equilibrium (t≫τ). The general solution can be written as 4 special forms, depending on whether the trap is initially occupied or empty at t=0 and whether it is occupied or empty after a time t:

(A3) $$P_{1}\left(t\right)=P+Qe^{-t/\tau };P_{1}\left(0\right)=1;\;(\mathrm{occupied}\;\mathrm{at}\;t=0;\;\mathrm{occupied}\;\mathrm{at}\;\mathrm{time}\;t)$$

(A4) $$Q_{1}\left(t\right)=Q-Qe^{-t/\tau };Q_{1}\left(0\right)=0;\;(\mathrm{occupied}\;\mathrm{at}\;t=0;\;\mathrm{empty}\;\mathrm{at}\;\mathrm{time}\;t)$$

(A5) $$P_{0}\left(t\right)=P-Pe^{-t/\tau };P_{0}\left(0\right)=0;\;(\mathrm{empty}\;\mathrm{at}\;t=0;\;\mathrm{occupied}\;\mathrm{at}\;\mathrm{time}\;t)$$

(A6) $$Q_{0}\left(t\right)=Q+Pe^{-t/\tau };Q_{0}\left(0\right)=1.\;(\mathrm{empty}\;\mathrm{at}\;t=0;\;\mathrm{empty}\;\mathrm{at}\;\mathrm{time}\;t)$$

Consider the CMS operation described by the timing diagram in Figure 2b with arbitrary $t_{cds}$ and $t_{d}$, the probability for each of 16 possible sampling outcomes can be calculated as a product of 4 factors on each row in Table A1. According to the approximate Equations (1) and (2), each sampling result generates one of five RTN noises: −ΔV, −ΔV/2, 0, ΔV/2,. It is straightforward to verify that the 16 probabilities add up to one with the help of $P_{1}\left(t\right)+Q_{1}\left(t\right)=1$ and $P_{0}\left(t\right)+Q_{0}\left(t\right)=1$.

**Table A1:** Probabilities of the 16 CMS sampling results.

SHR	SHR_d_	SHS	SHS_d_	RTN
P	$P_{1}\left(t_{d}\right)$	$Q_{1}\left(t_{cds}\right)$	$Q_{0}\left(t_{d}\right)$	ΔV
Q	$Q_{0}\left(t_{d}\right)$	$P_{0}\left(t_{cds}\right)$	$P_{1}\left(t_{d}\right)$	−ΔV
Q	$P_{0}\left(t_{d}\right)$	$P_{0}\left(t_{cds}\right)$	$P_{1}\left(t_{d}\right)$	−ΔV/2
P	$Q_{1}\left(t_{d}\right)$	$P_{1}\left(t_{cds}\right)$	$P_{1}\left(t_{d}\right)$	−ΔV/2
P	$P_{1}\left(t_{d}\right)$	$Q_{1}\left(t_{cds}\right)$	$P_{0}\left(t_{d}\right)$	ΔV/2
P	$P_{1}\left(t_{d}\right)$	$P_{1}\left(t_{cds}\right)$	$Q_{1}\left(t_{d}\right)$	ΔV/2
P	$Q_{1}\left(t_{d}\right)$	$Q_{1}\left(t_{cds}\right)$	$Q_{0}\left(t_{d}\right)$	ΔV/2
Q	$P_{0}\left(t_{d}\right)$	$Q_{0}\left(t_{cds}\right)$	$Q_{0}\left(t_{d}\right)$	ΔV/2
Q	$Q_{0}\left(t_{d}\right)$	$P_{0}\left(t_{cds}\right)$	$Q_{1}\left(t_{d}\right)$	−ΔV/2
Q	$Q_{0}\left(t_{d}\right)$	$Q_{0}\left(t_{cds}\right)$	$P_{0}\left(t_{d}\right)$	−ΔV/2
P	$P_{1}\left(t_{d}\right)$	$P_{1}\left(t_{cds}\right)$	$P_{1}\left(t_{d}\right)$	0
Q	$Q_{0}\left(t_{d}\right)$	$Q_{0}\left(t_{cds}\right)$	$Q_{0}\left(t_{d}\right)$	0
P	$Q_{1}\left(t_{d}\right)$	$P_{1}\left(t_{cds}\right)$	$Q_{1}\left(t_{d}\right)$	0
P	$Q_{1}\left(t_{d}\right)$	$Q_{1}\left(t_{cds}\right)$	$P_{0}\left(t_{d}\right)$	0
Q	$P_{0}\left(t_{d}\right)$	$Q_{0}\left(t_{cds}\right)$	$P_{0}\left(t_{d}\right)$	0
Q	$P_{0}\left(t_{d}\right)$	$P_{0}\left(t_{cds}\right)$	$Q_{1}\left(t_{d}\right)$	0

Consider the limiting case of $t_{cds}\gg \tau$, the $t_{d}$-dependent probability for each of the five RTN amplitudes, A(−ΔV), A(−ΔV/2), A(0), A(ΔV/2), A(ΔV), can be calculated from Table A1 as:

(A7) $$A\left(0\right)=P^{4}+Q^{4}+2PQe^{-t_{d}/\tau }\left(P^{2}+Q^{2}+PQe^{-t_{d}/\tau }\right)+4P^{2}Q^{2}{\left(1-e^{-t_{d}/\tau }\right)}^{2}$$

(A8) $$A\left(\Delta V\right)=A\left(-\Delta V\right)=PQ\left(P+Qe^{-t_{d}/\tau }\right)\left(Q+Pe^{-t_{d}/\tau }\right)$$

(A9) $$A\left(\Delta V/2\right)=A\left(-\Delta V/2\right)=2PQ\left(1-e^{-t_{d}/\tau }\right)\left(P^{2}+Q^{2}+2PQe^{-t_{d}/\tau }\right)$$

The cases of interests, CDS ($t_{d}=0$) and CMS ($t_{d}\gg \tau$), as shown in Figure 17a,b, can be readily derived as:

(A10) $$\mathrm{CDS}\;(t_{d}=0):\;A\left(0\right)=P^{2}+Q^{2};\;A\left(\Delta V/2\right)=0,\;A\left(\Delta V\right)=PQ;$$

(A11) $$\mathrm{CMS}\;(t_{d}\gg \tau ):\;A\left(0\right)=P^{4}+Q^{4}+4P^{2}Q^{2},\;A\left(\Delta V/2\right)=2PQ\left(P^{2}+Q^{2}\right),\;A\left(\Delta V\right)=P^{2}Q^{2}.$$

Finally, the $t_{d}$-dependent noise power ${\left(n_{RTN}\right)}^{2}$ can be calculated as the sum of the products of the probabilities and the square of the RTN terms in Table A1:

(A12) $${\left(n_{RTN}\right)}^{2}=\left[A\left(\Delta V/2\right)+A\left(-\Delta V/2\right)\right]{\left(\Delta V/2\right)}^{2}+\left[A\left(\Delta V\right)+A\left(-\Delta V\right)\right]{\left(\Delta V\right)}^{2}=PQ\left(1+e^{-t_{d}/\tau }\right){\left(\Delta V\right)}^{2},$$

which can be further reduced to the CDS case, ${\left(n_{RTN}\right)}^{2}=2PQ{\left(\Delta V\right)}^{2}$, when $t_{d}=0$ and the CMS case, ${\left(n_{RTN}\right)}^{2}=PQ{\left(\Delta V\right)}^{2}$, when $t_{d}\gg \tau$ as compared in Figure 17c. The derived $t_{d}$-dependency appears to account for the measured data versus $t_{d}$ in Figure 19b approximately. The factor-of-2 noise power reduction from CDS to CMS matches with the data as well. It is not difficult to rewrite Equations (A7) to (A12) more generally as functions of $t_{d}$ and $t_{cds}$ explicitly without assuming $t_{cds}\gg \tau$. Here we will not present the complete expressions, but it is straightforward to check out that, for CDS operation ($t_{d}=0$), the general formula will reduce to:

(A13) $${\left(n_{RTN}\right)}^{2}=2PQ\left(1-e^{-t_{cds}/\tau }\right){\left(\Delta V\right)}^{2}.$$

which is identical to the formula derived previously [20], i.e., Equation (A13) in [20].
